# RANKL confers protection against cell death in precision-cut lung slices

**DOI:** 10.3389/fphys.2022.1029697

**Published:** 2022-10-31

**Authors:** M. J. R. Ruigrok, M. A. P. Roest, H. W. Frijlink, P. Olinga, W. L. J. Hinrichs, B. N. Melgert

**Affiliations:** ^1^ Department of Pharmaceutical Technology and Biopharmacy, University of Groningen, Groningen, Netherlands; ^2^ Department of Molecular Pharmacology, University of Groningen, Groningen, Netherlands; ^3^ Groningen Research Institute of Asthma and COPD, University Medical Center Groningen, Groningen, Netherlands

**Keywords:** chronic bronchitis, emphysema, explant culture, OPGL, tissue regeneration, TRANCE

## Abstract

Chronic obstructive pulmonary disease (COPD) is the third leading cause of death globally and constitutes a major health problem. The disease is characterized by airflow obstructions due to chronic bronchitis and/or emphysema. Emerging evidence suggests that COPD is the result of impaired epithelial repair. Motivated by the need for more effective treatments, we studied whether receptor activator of nuclear factor κ-Β ligand (RANKL) contributed to epithelial repair, as this protein has been implicated in epithelial regeneration of breast and thymus. To do so, we used precision-cut lung slices prepared from mouse tissue—viable explants that can be cultured *ex vivo* for up to a few days while retaining features of lung tissue. Slices were cultured with 10, 100, or 500 ng/ml of mouse RANKL for 24 h. We first found RANKL activated nuclear factor κ-Β signaling, which is involved in cellular stress responses, without affecting the general viability of slices. Cell proliferation, however, was not altered by RANKL treatment. Interestingly, RANKL did reduce cell death, as revealed by TUNEL stainings and profiling of apoptosis-related proteins, indicating that it contributes to repair by conferring protection against cell death. This study improves our understanding of lung repair and could create new opportunities for developing COPD treatments.

## Introduction

Chronic obstructive pulmonary disease (COPD) has been estimated to be the third leading cause of death globally—surpassed only by ischemic heart disease and stroke—and constitutes a major health problem (WHO, 2020). The disease is characterized by airflow obstructions due to chronic bronchitis and/or emphysema. Tobacco smoking represents the most common cause of COPD. Other risk factors are indoor and outdoor air pollution, exposure to workplace fumes and dusts, and genetics (Hogea et al., 2020). COPD patients often display a reduced physical activity, which places them at risk for developing comorbidities, and they experience shortness of breath during exertion and eventually also at rest followed by respiratory failure (Barnes et al., 2015). Currently, COPD cannot be cured, and symptoms can only be partially relieved by using bronchodilators, antibiotics, and corticosteroids. Given the limited efficacy of currently available treatments, there remains a pressing medical need for more effective therapies.

The exact pathogenesis of COPD has not been fully elucidated, but recent evidence suggests that impaired epithelial repair plays an important role (Barnes, 2017). Restoring the regenerative capacity of progenitor cells, such as type II alveolar epithelial (ATII) cells, could therefore be a promising therapeutic approach for potentially reversing COPD. Out of all exogenous signaling molecules, receptor activator of nuclear factor κ-Β ligand (RANKL) is particularly interesting because it has been shown to stimulate epithelial cell proliferation in breast and thymus, suggesting a novel role for this protein (Ono et al., 2020). RANKL is a member of the tumor necrosis factor (TNF) family and typically binds to its target receptor RANK to control immune cell function and bone remodeling (Ono et al., 2020). We have recently discovered that intranasal administration of RANKL also increased the number of epithelial cells in the lungs of silica-treated mice (unpublished). The mechanism behind this effect, however, remains unclear.

The principal objective of this study was therefore to determine whether RANKL contributes to epithelial repair in lung tissue by stimulating cell proliferation and/or by reducing cell death. To that end, we used precision-cut lung slices, which are viable explants that can be cultured *ex vivo* for up to a few days while retaining functional and structural features of lung tissue (Ruigrok et al., 2021). Slices were prepared from mouse tissue, and they were cultured with soluble (mouse) RANKL at various concentrations for 24 h. We first investigated whether RANKL activated nuclear factor κ-Β (NF-κΒ) signaling and feedback mechanisms, after which we characterized the effects on cell proliferation and the number of ATII cells. We then examined RANKL’s effect on cell death and inflammation. As RANKL reduced cell death in slices, we also explored activation of pro-survival pathways and profiled expression of apoptosis-related proteins.

## Materials and methods

### Animals

Lungs were obtained from male C57BL/6J mice (8–12 weeks old; 24–30 G), which were housed under controlled conditions with free access to water and food. The mice were anesthetized using isoflurane/O_2_ (Nicolas Piramal, London, UK) and euthanized by exsanguination and puncturing of the diaphragm. To minimize warm ischemia, lungs were immediately inflated *in situ* with 1 ml of pre-warmed (37°C) and liquefied support medium, comprising 1.5% low gelling temperature agarose (Sigma-Aldrich, Zwijndrecht, Netherlands) and 0.9% NaCl (Merck, Darmstadt, Germany). The lungs were then swiftly resected and directly transferred to ice-cold Belzer University of Wisconsin (UW) Cold Storage Solution (Bridge-to-life, London, UK). The animal experiments were approved by the Central Authority for Scientific Procedures on Animals (permit number = 20171290) and were carried out in accordance with national and international legislation.

### Preparation of slices

Lung lobes were separated from each other, after which cylindrical tissue cores were made using a biopsy puncher. These tissue cores were immediately transferred to ice-cold UW Cold Storage Solution. Slices with a thickness of 250–300 µm and a diameter of 5 mm were prepared using a Krumdieck tissue slicer (Alabama Research and Development, Munford, USA), which was filled with ice-cold Krebs-Henseleit buffer supplemented with 25 mM D-glucose (Merck), 25 mM NaHCO_3_ (Merck), and 10 mM HEPES (MP Biomedicals, Aurora, USA); saturated with carbogen gas (95% O_2_ and 5% CO_2_); and adjusted to a pH of 7.4 (Ruigrok et al., 2021). After the slicing process, slices were directly transferred to ice-cold UW Cold Storage Solution.

### Incubation of slices

Slices were sampled directly after slicing (0 h) or pre-incubated individually in 12-well plates, containing pre-warmed (37°C) culture medium (1 ml/well), at 5% CO_2_ and 20% O_2_ while being horizontally shaken at 90 cycles/min. Culture medium consisted of Advanced DMEM/F-12 (ThermoFisher, Landsmeer, Netherlands) supplemented with 2 mM GlutaMAX (ThermoFisher), 10 mM HEPES (ThermoFisher), 100 U/mL Penicillin-Streptomycin (ThermoFisher), and 50 μg/ml Gentamicin (ThermoFisher). After a pre-incubation of 2 h, slices were transferred to culture plates with fresh and pre-warmed culture medium supplemented with (10, 100, or 500 ng/ml) or without (0 ng/ml) soluble mouse RANKL, which was produced as described previously (Wang et al., 2019). After an incubation of 24 h, slices were collected for analysis. Samples were obtained from four independent experiments (biological replicates), each carried out in triplicate (technical replicates).

### Enzyme-linked immunosorbent assay

Culture supernatants were analyzed with a Mouse OPG DuoSet ELISA (Bio-Techne, Abingdon, UK) and a Mouse RANKL DuoSet ELISA (Bio-Techne), according to the manufacturer’s instructions. Complexes of RANKL-OPG were analyzed in a modified procedure, using reagents from the same ELISAs (i.e., anti-RANKL capture antibodies and anti-OPG detection antibodies). The procedure has been previously described in detail (Mogi and Kondo, 2010). Optical densities were measured using a BioTek Synergy HT (BioTek Instruments, Vermont, USA), and wavelength correction was applied by subtracting readings at 540 nm from those at 450 nm.

### Proteome profiler antibody array

Semi-quantitative analysis of apoptosis-related proteins BAD, BCL2, BCL2L1, cleaved CASP3, CAT, CLSPN, CYC, FAS, HIF1A, HMOX1, HMOX2, HSP27, HSP60, HSP70, MCL1, KIP1, P53, SMAC, TNFRSF1A, TNFRSF10B, and XIAP was carried out with Proteome Profiler Mouse Apoptosis Arrays (Bio-Techne). For each experimental condition, samples were prepared by using equal amounts of protein lysate (100 µg). Subsequent steps were performed according to the manufacturer’s instructions. The protein dots were simultaneously visualized using Clarity Western ECL Substrate (Bio-Rad, Lunteren, Netherlands) and a ChemiDoc Touch Imaging System (Bio-Rad). To allow for visualization in heatmaps, row z-scores were calculated, using the formula: z = (x - μ)/σ, wherein x is the raw score, μ is the population mean, and σ is the population standard deviation. A positive row z-score indicates expression was higher than the mean, whereas a negative one reflected expression levels below the mean.

### Quantitative real-time polymerase chain reaction

Slices were first lysed through mechanical disruption, after which total RNA was isolated with a Maxwell 16 LEV SimplyRNA Tissue Kit (Promega, Leiden, Netherlands). The isolated RNA (300 ng) was then reverse transcribed using a Reverse Transcription System Kit (Promega) and thermal cycler (10 min at 22°C, 15 min at 42°C, and 5 min at 95°C). After synthesis, the cDNA concentration was 15 ng/μL. Quantitative real-time polymerase chain reaction (qPCR) was conducted using specific primers (Supplementary Table S1), FastStart universal SYBR Green Master Mix (Roche, Almere, Netherlands), and a ViiA7 qPCR system (Applied Biosystems, Bleiswijk, Netherlands). The qPCR system was configured with 1 cycle of 10 min at 95°C and 40 cycles of 15 s at 95°C, 30 s at 60°C, and 30 s at 72°C. To that end, 384-well plates were used, and each well contained 5 μL of SYBR Green Master Mix (incl. 5 μM of forward and reverse primers) and 5 μL of cDNA at a concentration of 1 ng/μL. The primers were validated *in silico* using Primer-BLAST software, after which amplification efficiencies were tested *in vitro* by preparing 8-point standard curves. Amplification efficiencies between 90 and 110% were considered acceptable. The melting curves were also checked to confirm single amplicons were produced. We calculated relative gene expression with the two to the power of -ΔCt (2-ΔCt) method, using *18s* for normalization purposes.

### Stainings

Slices were fixed in 4% buffered formalin, embedded in paraffin, and cut into 4 µm sections. The tissue morphology was visualized using routine hematoxylin and eosin (H&E) stainings, and DNA fragmentation was revealed through the use of an HRP-DAB TUNEL Assay Kit (Abcam, Cambridge, USA). In addition, proliferating cells and ATII cells were visualized by immunohistochemical staining of KI67 and SFTPC, respectively. Briefly, sections were subjected to heat-mediated (80°C) antigen retrieval in Diva Decloaker (Biocare Medical, Pacheco, USA) for 15 min, blocked with Background Punisher (Biocare Medical) for 5 min, incubated with either rabbit anti-KI67 (ab15580, 1:750, Abcam) or rabbit anti-SFTPC (ab90716, 1:750, Abcam) for 1 h, incubated with MACH 4 universal HRP-Polymer (Biocare Medical) for 30 min, incubated with Betazoid DAB Chromogen (Biocare Medical) for 15 min, counterstained with hematoxylin for 1–2 s, and mounted with DEPEX (Sigma-Aldrich). Stained sections were scanned using a C9600 NanoZoomer (Hamamatsu Photonics, Hamamatsu, Japan). The KI67 and TUNEL staining intensity was quantified with Aperio ImageScope (V12.3.3), using the Aperio Positive Pixel Count Algorithm (V9). For each sample (biological replicate), three entire slices (technical replicates) were analyzed to count negative, weak positive, medium positive, and strong positive pixels. The staining intensity was calculated as the ratio of strong positive pixels *vs.* total pixels.

### Viability assay

Slice viability was assessed by measuring adenosine triphosphate (ATP) levels. Slices were first homogenized by means of mechanical disruption, as described previously (Ruigrok et al., 2021). Supernatants were subsequently analyzed using an ATP Bioluminescence Assay CLS II Kit (Sigma-Aldrich). The same samples were incubated overnight at 37°C with open lids to remove homogenization solution through evaporation. The protein content of the leftover pellet was measured with an RC DC Protein Assay Kit (Bio-Rad). Obtained ATP levels (pmol) were normalized to protein content (µg).

### Western blotting

Slices were lysed using RIPA Lysis Buffer (ThermoFisher) supplemented with Halt Protease Inhibitor Cocktail (ThermoFisher) and Halt Phosphatase Inhibitor Cocktail (ThermoFisher). The protein content in lysates was then determined with a Pierce BCA Protein Assay Kit (ThermoFisher). Extracted protein (10 µg/lane) was subsequently separated through sodium dodecyl sulfate polyacrylamide gel electrophoresis using 4–15% Mini-PROTEAN TGX Precast Protein Gels (Bio-Rad), and blotted onto membranes using a Trans-Blot Turbo RTA Mini 0.2 µm PVDF Transfer Kit (Bio-Rad). After blocking in 5% non-fat dry milk/TBST (Bio-Rad) for 1 h, membranes were incubated overnight at 4°C with primary antibodies (Supplementary Table S2), followed by an incubation with HRP-conjugated secondary antibodies for 1 h. Thereafter, protein bands were visualized using Clarity Western ECL Substrate (Bio-Rad) and a ChemiDoc Touch Imaging System (Bio-Rad). To evaluate the fraction of phosphorylated protein, we first detected the levels of phosphorylated protein (i.e., p-PI3K and p-AKT) and then stripped membranes using Restore Western Blot Stripping Buffer (ThermoFisher), after which we detected the corresponding total levels of protein (i.e., t-PI3K and t-AKT). In other cases, vinculin (VCL) was used as a loading control.

### Statistics

Prism for macOS version 9.3.1. (GraphPad Software, San Diego, USA) was used to conduct statistical data analysis. To compare means from treatment groups with a single control group, we analyzed data using an ordinary one-way analysis of variance (ANOVA) followed by Dunnett’s multiple comparisons test. To detect culture-associated changes (i.e., between untreated slices at 0 and 24 h), we performed unpaired two-tailed *t* tests. Differences were statistically significant when *p* < 0.05.

## Results

### Slice viability and morphology

**FIGURE 1:**
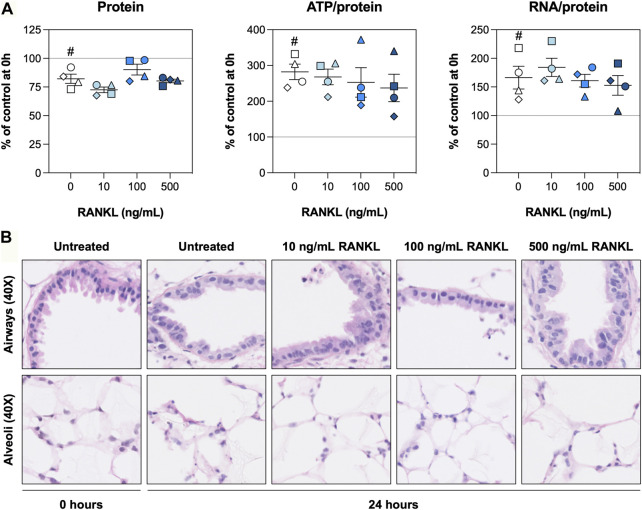
Slice viability and morphology. Slices were sampled after slicing (0 h) and after 24 h of incubation without or with 10, 100, or 500 ng/ml RANKL (*n* = 4). The protein, ATP/protein, and RNA/protein content **(A)** was determined to assess general slice viability, and H&E stainings **(B)** were carried out to visualize the morphology. Values represent individual experiments performed in triplicate and are accompanied with the arithmetic mean (horizontal line) ± standard error of the mean (error bars). (^#^ indicates *p* < 0.05 between untreated slices at 0 and 24 h).

To find out whether slices remained viable during the experiments, we first assessed the protein, ATP/protein, and RNA/protein content as well as the morphology. The protein content was slightly reduced after 24 h and remained unaffected by RANKL (Figure 1A). The ATP/protein and RNA/protein content, however, was increased after 24 h, although no significant differences were observed for slices treated with RANKL. The H&E stainings revealed the morphology of airways and alveoli remained well-preserved for 24 h, with only minor signs of tissue damage (Figure 1B). Low magnification figures of H&E stainings are shown in Supplementary Figure S1.

### NF-κΒ signaling

**FIGURE 2:**
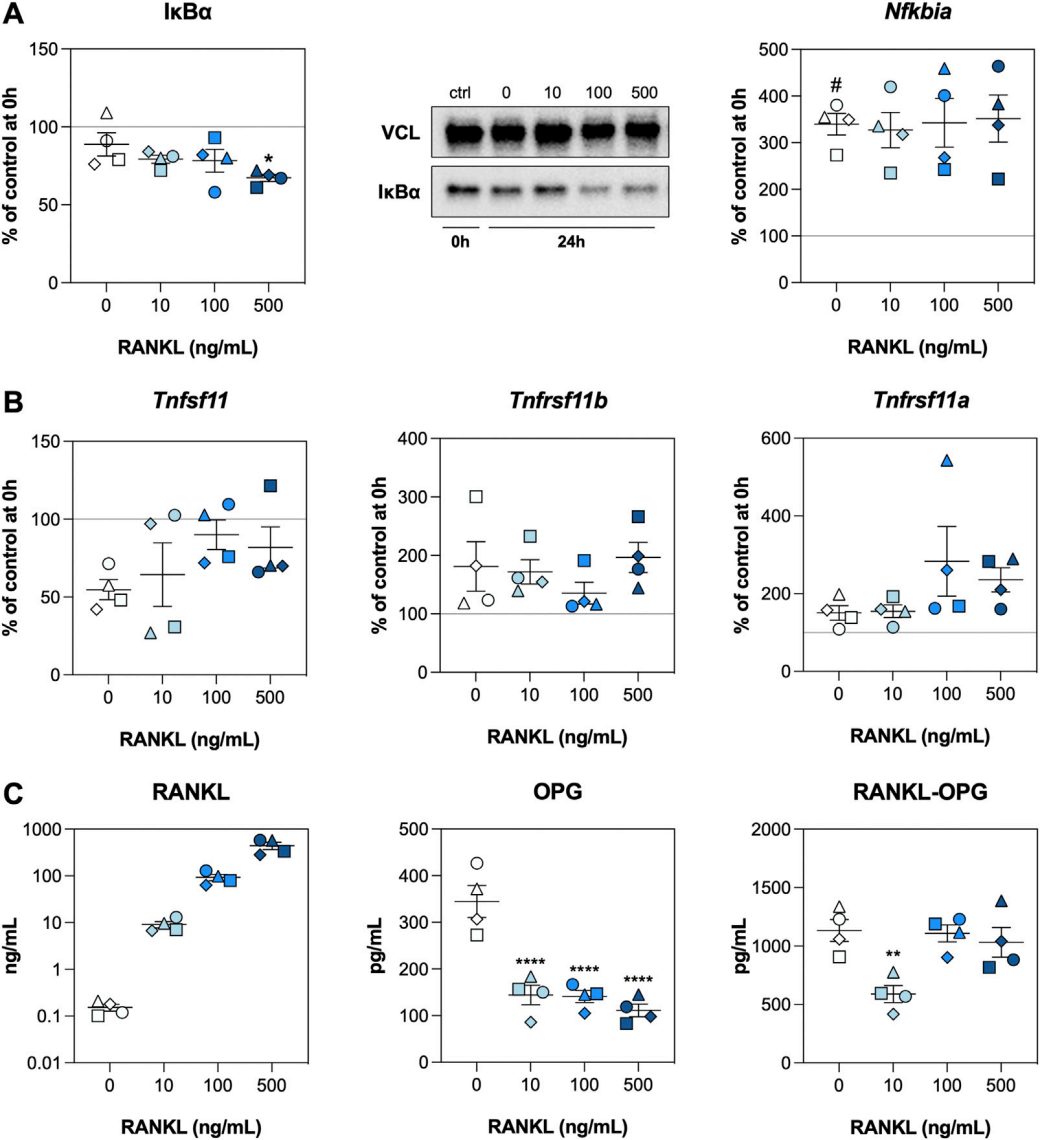
NF-κB signaling. Slices were sampled after slicing (0 h) and after 24 h of incubation without or with 10, 100, or 500 ng/ml RANKL (*n* = 4). Expression of IκBα and *Nfkbia* **(A)**, expression of *Tnfsf11*, *Tnfrsf11b*, and *Tnfrsf11a* **(B)**, and concentrations of RANKL, OPG, and RANKL-OPG **(C)** were analyzed to detect activation of NF-κΒ signaling. Values represent individual experiments performed in triplicate and are accompanied with the arithmetic mean (horizontal line) ± standard error of the mean (error bars). (^#^ indicates *p* < 0.05 between untreated slices at 0 and 24 h; *, **, and **** indicate *p* < 0.05, <0.01, and <0.0001, respectively, between untreated slices and slices treated with exogenous RANKL at 24 h).

We subsequently investigated whether RANKL activated NF-κΒ signaling and potential feedback mechanisms by analyzing inhibitor of nuclear factor κ-Β-α (IκBα) content as well as mRNA and protein levels of related factors. The IκBα content remained stable after an incubation of 24 h when no exogenous RANKL was added, but a significant reduction was observed for slices treated with RANKL at 500 ng/ml (Figure 2A). We did, however, observe that mRNA expression of *Nfkbia* (encodes IκBα) was increased after 24 h, regardless of the RANKL concentration. Subsequent analyses revealed that mRNA expression of *Tnfsf11* (encodes RANKL) was reduced after 24 h of incubation, whereas that of *Tnfrsf11b* (encodes OPG) and *Tnfrsf11a* (encodes RANK) increased (Figure 2B). Having said that, expression of these genes remained unaffected by RANKL. Next, the concentrations of RANKL matched the amount added, and levels of OPG were significantly reduced by RANKL, down to a minimum of ∼125 pg/ml (Figure 2C). Lastly, RANKL-OPG complex levels were significantly reduced when slices were treated with RANKL at 10 ng/ml.

### Cell proliferation

**FIGURE 3:**
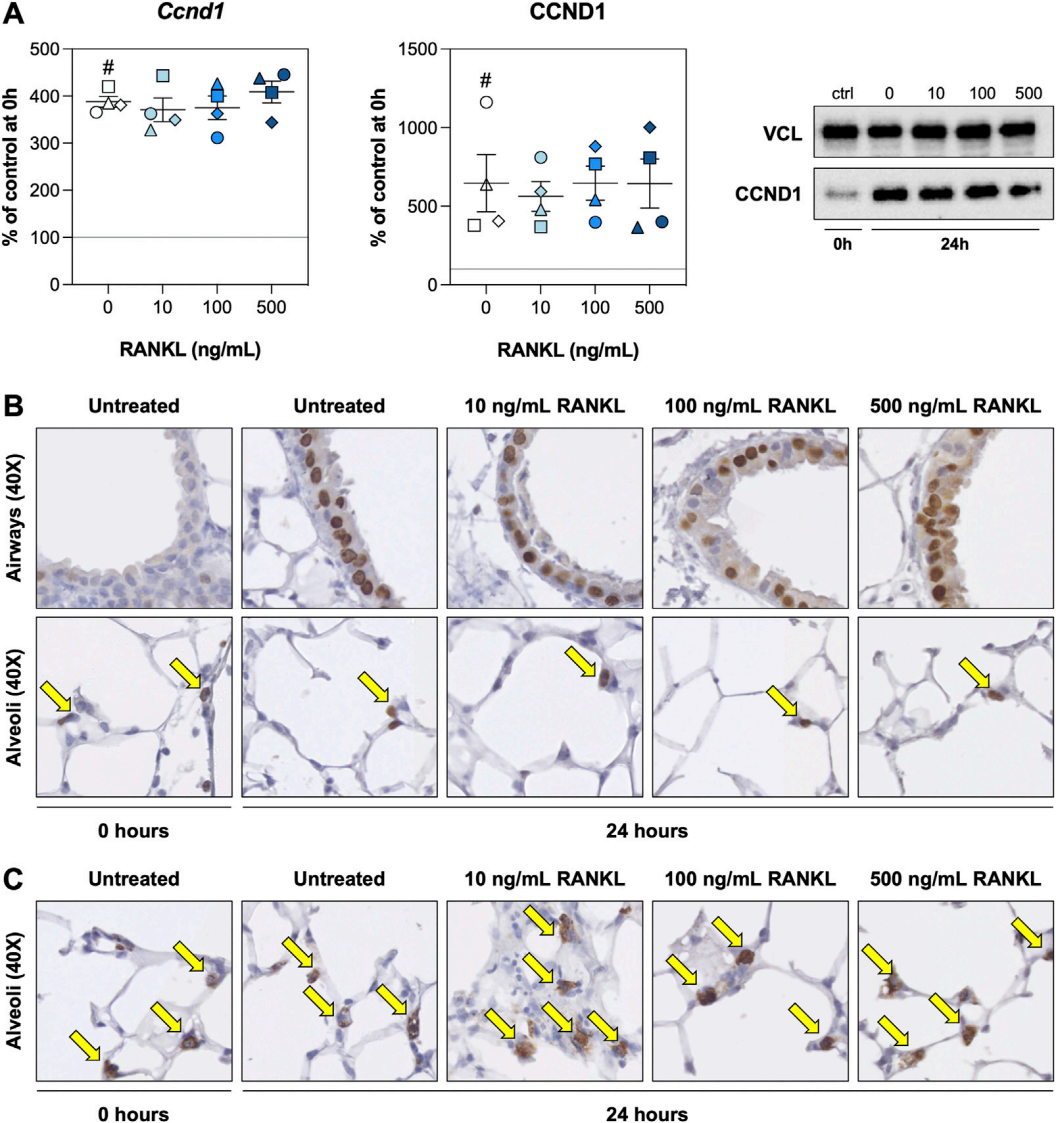
Cell proliferation. Slices were sampled after slicing (0 h) and after 24 h of incubation without or with 10, 100, or 500 ng/ml RANKL (*n* = 4). Cyclin D1 mRNA and protein expression **(A)** were analyzed to assess whether cell proliferation was affected by RANKL. To visualize proliferating cells and ATII cells (arrows), we stained sections for KI67 **(B)** and SFTPC **(C)**, respectively. Values depict individual experiments performed in triplicate and are accompanied with the arithmetic mean (horizontal line) ± standard error of the mean (error bars). (^#^ indicates *p* < 0.05 between untreated slices at 0 and 24 h).

We hypothesized that RANKL could promote cell proliferation. We therefore examined expression of cyclin D1—both on mRNA and protein level—and visualized proliferating cells and ATII cells by staining for KI67 and SFTPC, respectively. As demonstrated, the expression of *Ccnd1* mRNA, as well as its functional gene product CCND1, were increased after 24 h of culture but remained unaffected by RANKL treatment (Figure 3A). Stainings for KI67 revealed that proliferating cells in the airways were significantly more abundant after 24 h of incubation (Supplementary Figure S2), though this increase seemed to become less pronounced (not significant) at increasing RANKL concentrations (Figure 3B). KI67 expression in the alveolar region, however, remained unchanged during culture or when RANKL was added. Likewise, the number of ATII cells remained stable during culture and was not affected by RANKL treatment, as determined by SFTPC stainings (Figure 3C). Low magnification figures of KI67 and SFTPC stainings are shown in Supplementary Figures S2, S3, respectively.

### Cell death and inflammation

**FIGURE 4:**
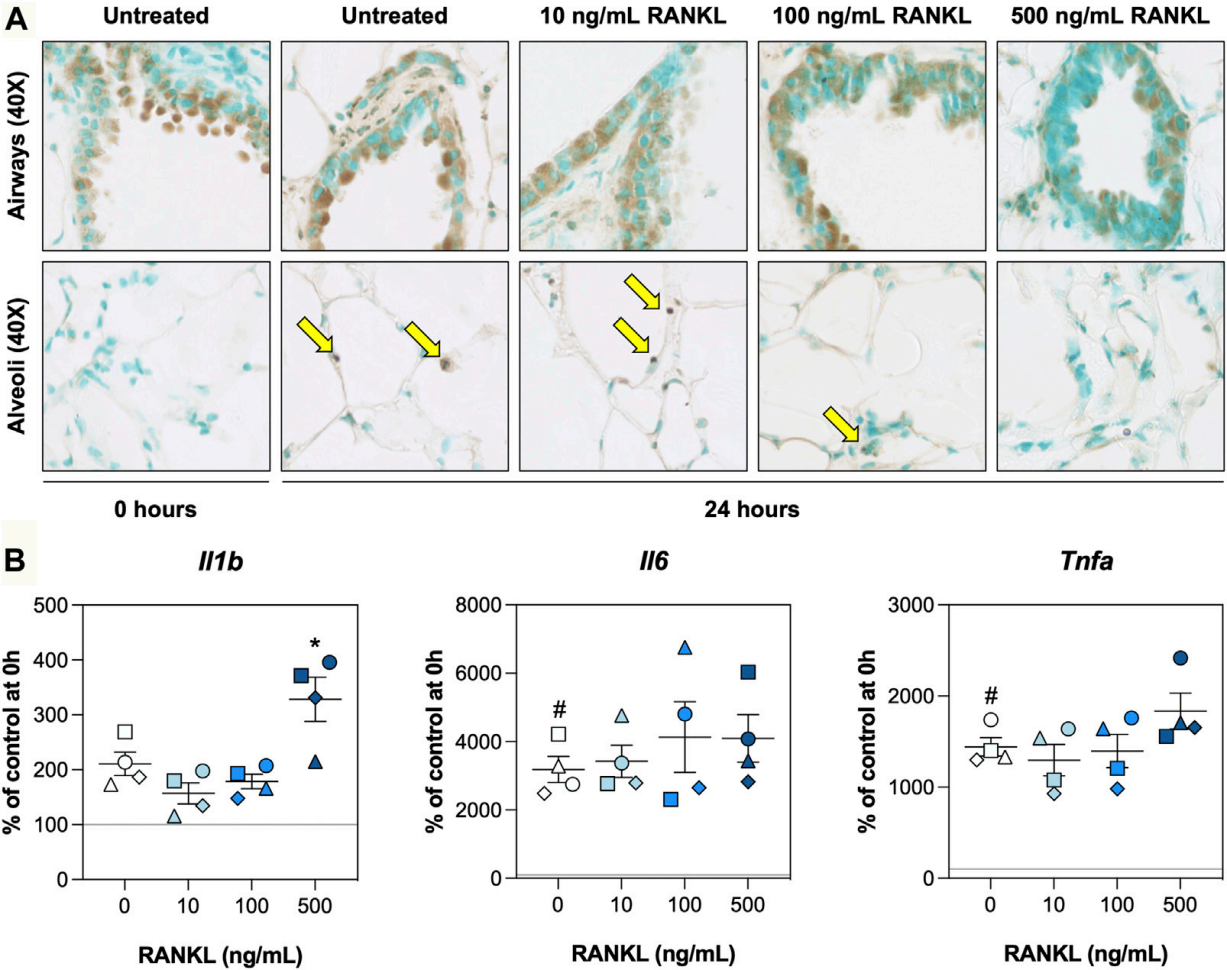
Cell death and inflammation. Slices were sampled after slicing (0 h) and after 24 h of incubation without or with 10, 100, or 500 ng/ml RANKL (*n* = 4). TUNEL stainings **(A)** were carried out to visualize DNA fragmentation (arrows), and mRNA expression of pro-inflammatory cytokines **(B)** was measured to study inflammation. Values represent individual experiments performed in triplicate and are accompanied with the arithmetic mean (horizontal line) ± standard error of the mean (error bars). (^#^ indicates *p* < 0.05 between untreated slices at 0 and 24 h; * indicates *p* < 0.05 between untreated slices and slices treated with exogenous RANKL at 24 h).

To determine whether RANKL mitigated cell death and/or inflammation, we visualized DNA fragmentation using TUNEL stainings and characterized mRNA expression of pro-inflammatory cytokines. TUNEL stainings revealed that, directly after slicing, DNA fragmentation was mostly confined to the airways, with only very few instances in the alveoli (Figure 4A). Culturing slices for 24 h resulted in more DNA fragmentation in alveoli. However, slices treated with 500 ng/ml RANKL displayed significantly less DNA fragmentation in both airways and alveoli (Supplementary Figure S4). Low magnification figures of TUNEL stainings are shown in Supplementary Figure S4. Similar culture-associated observations were made for mRNA expression of *Il1b*, *Il6*, and *Tnfa*, as their expression was markedly increased after 24 h of incubation (Figure 4B). At the highest concentration, RANKL only significantly increased mRNA expression of *Il1b*.

### PI3K/Akt signaling and apoptosis

**FIGURE 5:**
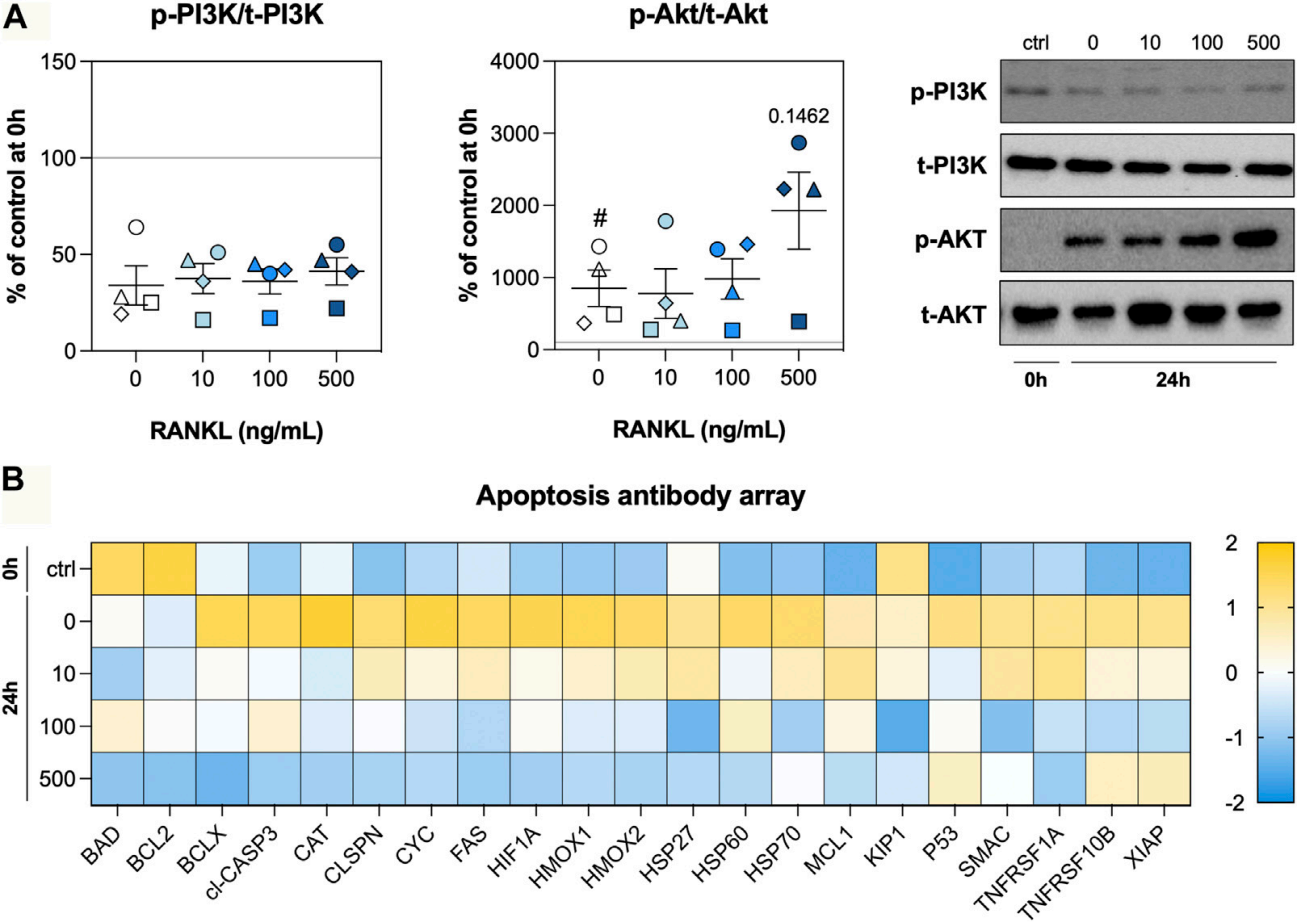
PI3K/Akt signaling and apoptosis. Slices were sampled after slicing (0 h) and after 24 h of incubation without or with 10, 100, or 500 ng/ml RANKL (*n* = 4). The phosphorylation of PI3K and Akt **(A)** was analyzed to determine whether the tissue adopted a pro-survival phenotype. We profiled apoptosis-related proteins **(B)** to obtain more information about stressors and effectors (row z-score). Values represent individual experiments performed in triplicate and are accompanied with the arithmetic mean (horizontal line) ± standard error of the mean (error bars). (^#^ indicates *p* < 0.05 between untreated slices at 0 and 24 h).

As RANKL treatment resulted in less DNA fragmentation, we assessed activation of the PI3K/Akt signaling pathway and profiled the expression of a large number of apoptosis-related proteins. After 24 h of incubation, phosphorylation of PI3K was markedly reduced regardless of the RANKL concentration, whereas phosphorylation of Akt became more prominent during culture (Figure 5A). At 500 ng/ml, RANKL seemed to enhance phosphorylation of Akt in slices, albeit not significantly. Subsequent profiling of apoptosis-related proteins revealed higher expression levels in untreated slices that were cultured for 24 h (Figure 5B). For most of the tested proteins, these increased expression levels were attenuated by RANKL in a seemingly concentration-dependent manner. In fact, when slices were treated with 500 ng/ml RANKL, levels of some proteins were restored to those observed directly after slicing.

## Discussion

The goal of this study was to find out whether RANKL contributes to epithelial repair in lung tissue by stimulating cell proliferation and/or by reducing cell death. Our study demonstrated that RANKL treatment successfully activated the NF-κB signaling pathway without affecting the viability of slices. Surprisingly, RANKL treatment slightly reduced cell proliferation in the airways, whereas the alveolar region was not affected, even though SFTPC stainings revealed ATII cells were abundant. Cell death, however, was strongly reduced by RANKL, as revealed by TUNEL stainings and profiling of apoptosis-related proteins. Our findings therefore indicate RANKL contributes to epithelial repair in lung tissue by conferring protection against cell death.

To get to these findings, we first analyzed the protein, ATP/protein, and RNA/protein content as well as morphology to monitor the overall health of slices. The protein content, for example, was lower after 24 h of incubation. This phenomenon has been observed previously, albeit for longer incubation times (48 h), and probably reflects a loss of cells due to apoptosis and necrosis (Ruigrok et al., 2018; Ruigrok et al., 2019; Ruigrok et al., 2021). Additionally, ATP/protein levels were higher after the incubation, owing to the fact that slices are subjected to warm and cold ischemia prior to their incubation (Chen-Yoshikawa, 2021). The RNA/protein content was slightly increased after culture, possibly due to elevated transcription of genes required for tissue repair (e.g., cytokines and matrix proteins) (Kotton and Morrisey, 2014). Lastly, H&E stainings revealed minor signs of tissue damage, which is in line with our previous observations, allowing us to explore anti-apoptotic effects of RANKL. This would not have been possible in the absence of cell death. These findings indicate the slices remained sufficiently viable for subsequent experiments and analyses.

The next step was to determine whether RANKL activated NF-κB signaling and potential feedback mechanisms. As shown, RANKL treatment resulted in significantly lower IκBα levels. This finding confirms that the NF-κB signaling pathway was activated, as binding of RANKL to RANK triggers proteasomal degradation of IκBα through activation of IκB kinase (IKK), enabling translocation of NF-κB (Liu et al., 2017; Infante et al., 2019). We also observed the activation of a negative feedback loop, as *Nfkbia* mRNA expression was higher after incubation, regardless of the RANKL concentration (Dorrington and Fraser, 2019). The lack of a concentration-dependency seems to indicate saturation of this negative feedback loop, which aims to terminate NF-κB signaling. Interestingly, RANKL significantly reduced the secretion of OPG into culture medium. The cause of this effect, however, remains unclear as mRNA expression of *Tnfrsf11b* remained unaffected by RANKL and RANKL-OPG complex levels were quite stable. These findings suggest RANKL could have affected the translational efficiency, the secretion kinetics of OPG, or the uptake/degradation of RANKL-OPG complexes.

We then studied the effect of RANKL on cell proliferation. Surprisingly, RANKL did not affect cyclin D1 mRNA and protein levels. This was not expected because NF-κB activation has been shown to be required for optimal cyclin D1 induction in mammary epithelial cells (Cao et al., 2001; Infante et al., 2019). Stainings for KI67 corroborated this finding as we found no changes in Ki67 expression when slices were treated with RANKL (Sobecki et al., 2017). The airways, however, contained fewer proliferating cells at increasing RANKL concentrations. Cell proliferation in the airways greatly increased during culture, whereas it remained at a steady state in the alveolar region, seemingly unaffected by the incubation. To rule out that the lack of effects in the alveolar region was caused by changes in the number of ATII cells, which act as progenitor cells for type I alveolar epithelial cells, we stained sections for SFTPC (Zepp and Morrisey, 2019). This staining revealed the number of ATII cells remained stable over time, unaffected by RANKL treatment. Therefore, it might have been the case ATII cells were not switching to a proliferative phenotype due to the incubation time being too short, as slices were cultured for only 24 h, or because of lacking growth factors and/or other signals (Barkauskas et al., 2017). In the future, it would be interesting to test the effects of RANKL over an extended period of time (e.g., for up to 7 days, taking samples every 24 h), though care should be taken to characterize culture-associated changes to progenitor and supporting cells to ensure their complex interactions are captured and not lost due to cell death.

After finding out that cell proliferation was not affected by RANKL, we explored its effect on cell death. To do so, we carried out TUNEL stainings to detect DNA fragmentation—a key feature of apoptosis but also, in some cases, necrosis (Kraupp et al., 1995). The stainings revealed RANKL substantially reduced cell death, both in the airways and alveolar region. This finding is in line with previously reported effects that were observed upon activation of NF-κB signaling, as this pathway not only regulates inflammation but also cell survival (Liu et al., 2017). We suspect this reduction in cell death averted the need for cell proliferation in the airways (i.e., less dead or dying cells had to be replaced by new cells) (Kotton and Morrisey, 2014). This could explain why RANKL did not promote proliferation in the alveolar region, as fairly low numbers of dying cells were observed. We also showed that 500 ng/ml RANKL increased mRNA expression of *Il1b*, which is one of the target genes controlled by NF-κB, further suggesting that cells attained a pro-survival phenotype (Galluzzi et al., 2018; Ono et al., 2020). It remains unclear, however, whether effects were the same for each cell type.

To get more insight into the pro-survival phenotype induced by RANKL, we investigated PI3K/Akt signaling and profiled apoptosis-related proteins. Surprisingly, the activation of PI3K signaling was strongly reduced after 24 h of incubation, whereas Akt signaling was more profound. This discrepancy indicates Akt was likely activated in a PI3K-independent manner, perhaps by tyrosine or serine/threonine kinases Ack1, Src, PTK6, TBK1, or IKBKE (Mahajan and Mahajan, 2012). The profiling of apoptosis-related proteins revealed RANKL normalized their expression in a concentration-dependent manner, thereby corroborating our previous observations with the TUNEL stainings because we detected less cell death. Of particular interest is the normalization of cleaved CASP3 levels—an effector caspase that digests intracellular proteins during the execution-phase of apoptosis (Galluzzi et al., 2018). On top of that, levels of XIAP, which is one of the most potent inhibitors of apoptosis, was higher in slices that were incubated with 500 ng/ml of RANKL (Fullerton and Gilroy, 2016). These observations therefore demonstrate that RANKL contributes to epithelial repair in lung tissue by reducing cell death.

While this study sheds a new light on the role of RANKL in controlling life and death in lung tissue, it also has some limitations. The lacking recruitment of immune cells (e.g., lymphocytes, neutrophils, monocytes etc.) as well as species differences in epithelial biology between mice and humans make it difficult to translate our findings to COPD patients (Barnes, 2017; Tashiro et al., 2017; Zepp and Morrisey, 2019). As a next step, it would be useful to study the effects of RANKL in lung slices exposed to various pathological stimuli (e.g., cigarette smoke extract and/or elastase), after which effects could be further validated in slices prepared from explanted lung tissue of COPD patients (Liu et al., 2019). Additionally, because of the analytical techniques used, we were unable to discern cell-specific effects. Leveraging technological innovations, such as spatial transcriptomics and single-cell sequencing, could provide valuable insights into cell-specific responses (Travaglini et al., 2020). Our findings also raise a more fundamental question regarding the therapeutic application of RANKL, as apoptosis-modulating drugs remain controversial due to potential risks of tumorigenesis. Follow-up studies should address whether RANKL is capable of overriding the intracellular machinery responsible for removing dangerous cells (e.g., P53-mediated apoptosis of cancer cells) (Dashzeveg and Yoshida, 2015).

## Conclusion

The aim of this work was to determine whether RANKL contributes to epithelial repair in the lungs by enhancing cell proliferation and/or by reducing cell death. We first demonstrated that RANKL activated the NF-κB signaling pathway as well as a negative feedback loop without affecting the viability of slices. We then showed RANKL seemed to slightly reduce cell proliferation in the airways, although other areas remained unaffected. Cell death, however, was significantly reduced by RANKL, both in the airways and alveolar region. Our study therefore suggests that RANKL contributes to epithelial repair in the lungs by conferring protection against cell death. These findings improve our understanding of lung repair and could open new opportunities for the development of treatments for COPD patients.

## Data Availability

The raw data supporting the conclusions of this article will be made available by the authors, without undue reservation.

## Glossary

ANOVA: analysis of variance
ATII: type 2 alveolar epithelial
ATP: adenosine triphosphate
BAD: B-cell lymphoma 2 associated agonist of cell death
BCL2: B-cell lymphoma 2
BCL2L1: B-cell lymphoma 2 like 1
CASP3: caspase 3
CAT: catalase
CLSPN: claspin
COPD: chronic obstructive pulmonary disease
CYC: cytochrome c
FAS: fas cell surface death receptor
H&E: hematoxylin and eosin
HIF1A: hypoxia inducible factor 1 subunit alpha
HMOX1: heme oxygenase 1
HMOX2: heme oxygenase 2
HSP27: heat shock protein 27
HSP60: heat shock protein 60
HSP70: heat shock protein 70
IkBα: inhibitor of nuclear factor κ-Β-α
IKK: inhibitor of nuclear factor κ-Β kinase
KIP1: cyclin dependent kinase inhibitor 1B
MCL1: myeloid cell leukemia 1
NF-κΒ: nuclear factor κ-Β
OPG: osteoprotegerin
P53: tumor protein p53
qPCR: quantitative real-time polymerase chain reaction
RANKL: receptor activator of nuclear factor κ-Β ligand
SMAC: diablo IAP-binding mitochondrial protein
TNF: tumor necrosis factor
TNFRSF10B: tumor necrosis factor receptor superfamily member 10B
TNFRSF1A: tumor necrosis factor receptor superfamily member 1A
UW: University of Wisconsin
VCL: vinculin
XIAP: X-linked inhibitor of apoptosis.
