# Supplementary material for: RANKL confers protection against cell death in precision-cut lung slices
Source: Front Physiol. 2022 Oct 31;13:1029697. doi: 10.3389/fphys.2022.1029697 (PMC9659591; doi:10.3389/fphys.2022.1029697)
Supplement: Supplementary file 1 [file DataSheet1.PDF]

Supplementary information

RANKL confers protection against cell death in precision-cut lung slices

M.J.R. Ruigrok[1], M.A.P. Roest[1], H.W. Frijlink[1], P. Olinga[1], W.L.J. Hinrichs[1], B.N. Melgert[2,3*]

[1] Department of Pharmaceutical Technology and Biopharmacy, University of Groningen, Antonius Deusinglaan 1, 9713 AV Groningen, The Netherlands.

[2] Department of Molecular Pharmacology, University of Groningen, Antonius Deusinglaan 1, 9713 AV Groningen, The Netherlands.

[3] Groningen Research Institute of Asthma and COPD, University Medical Center Groningen, Hanzeplein 1, 9713 GZ Groningen, The Netherlands.

[*] Corresponding author (phone number: +31 (0)50 363 2947, e-mail address: b.n.melgert@rug.nl).

Supplementary table 1. Primers.

Gene	Protein	Forward sequence (5' → 3')	Reverse sequence (5' → 3')
18s	-	CTTAGAGGGACAAGTGGCG	ACGCTGAGCCAGTCAGTGTA
Ccna2	CCNA2	AAGAGAATGTCAACCCCGAAAAA	ACCCGTCGAGTCTTGAGCTT
Ccnb1	CCNB1	CTTGCAGTGAGTGACGTAGAC	CCAGTTGTCGGAGATAAGCATAG
Ccnd1	CCND1	GCGTACCCTGACACCAATCTC	ACTTGAAGTAAGATACGGAGGGC
Ccne1	CCNE1	CTCCGACCTTTCAGTCCGC	CACAGTCTTGTCAATCTTGGCA
Il1b	IL-1β	TGAGCACCTTCTTTTCCTTCA	TTGTCTAATGGGAACGTCACAC
Il6	IL-6	TGATGCTGGTGACAACCACGGC	TAAGCCTCCGACTTGTGAAGTGGTA
Nfkbia	IκBα	CCTGACCTGGTTTCGCTCTT	AGGTAAGCTGGTAGGGGGAG
Tnfa	TNF-α	CTGTAGCCCACGTCGTAGC	TTGAGATCCATGCCGTTG
Tnfrsf11a	RANK	CCAGGAGAGGCATTATGAGCA	ACTGTCGGAGGTAGGAGTGC
Tnfrsf11b	OPG	ACAGTTTGCCTGGGACCAAA	CTGTGGTGAGGTTCGAGTGG
Tnfsf11	RANKL	CCTGTACTTTCGAGCGCAGA	GCATTGATGGTGAGGTGTGC

Supplementary table 2. Antibodies.

Protein	Primary antibody	Secondary antibody
VCL	Mouse anti-VCL<br>(sc73614, 1:500, Santa Cruz)	Rabbit anti-mouse immunoglobins/HRP<br>(P0260, 1:5000, Dako)
IκBα	Rabbit anti-IκBα<br>(ab32518, 1:2500, Abcam)	Goat anti-rabbit immunoglobins/HRP<br>(P0448, 1:2000, Dako)
CCND1	Rabbit anti-CCND1<br>(ab134175, 1:10000, Abcam)	Goat anti-rabbit immunoglobins/HRP<br>(P0448, 1:2000, Dako)
p-PI3K	Rabbit anti-phospho-PI3K<br>(ab182651, 1:1000, Abcam)	Goat anti-rabbit immunoglobins/HRP<br>(P0448, 1:2000, Dako)
t-PI3K	Rabbit anti-total-PI3K<br>(ab191606, 1:1000, Abcam)	Goat anti-rabbit immunoglobins/HRP<br>(P0448, 1:2000, Dako)
p-Akt	Rabbit anti-phospho-Akt<br>(ab192623, 1:1000, Abcam)	Goat anti-rabbit immunoglobins/HRP<br>(P0448, 1:2000, Dako)
t-Akt	Rabbit anti-total-Akt<br>(ab179463, 1:5000, Abcam)	Goat anti-rabbit immunoglobins/HRP<br>(P0448, 1:2000, Dako)

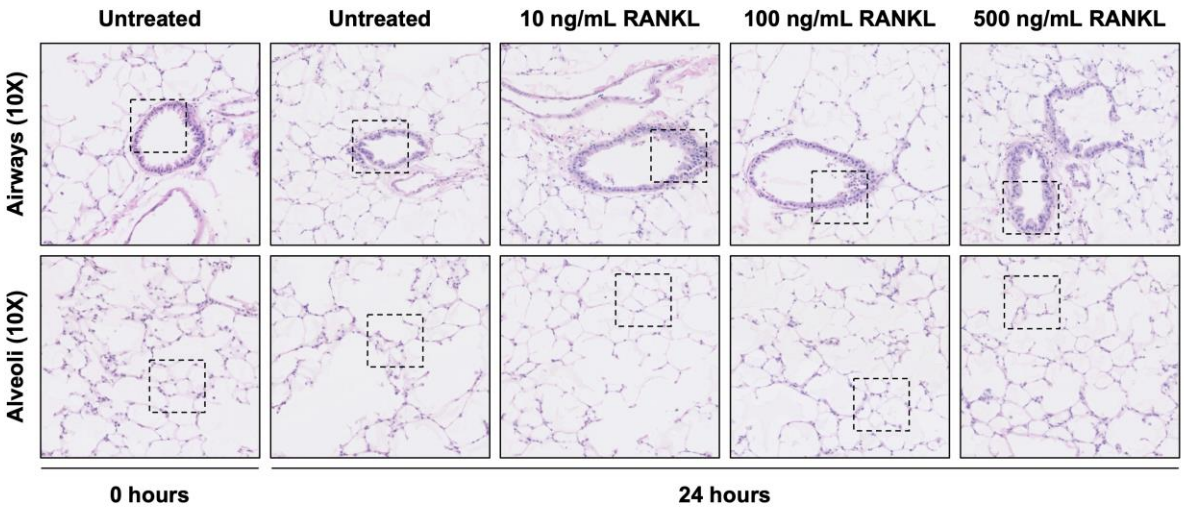

Supplementary figure S1. H&E stainings. Slices were sampled after slicing (0 h) and after 24 h of incubation without or with 10, 100, or 500 ng/mL RANKL (*n* = 4). H&E stainings were subsequently carried out to visualize the morphology.

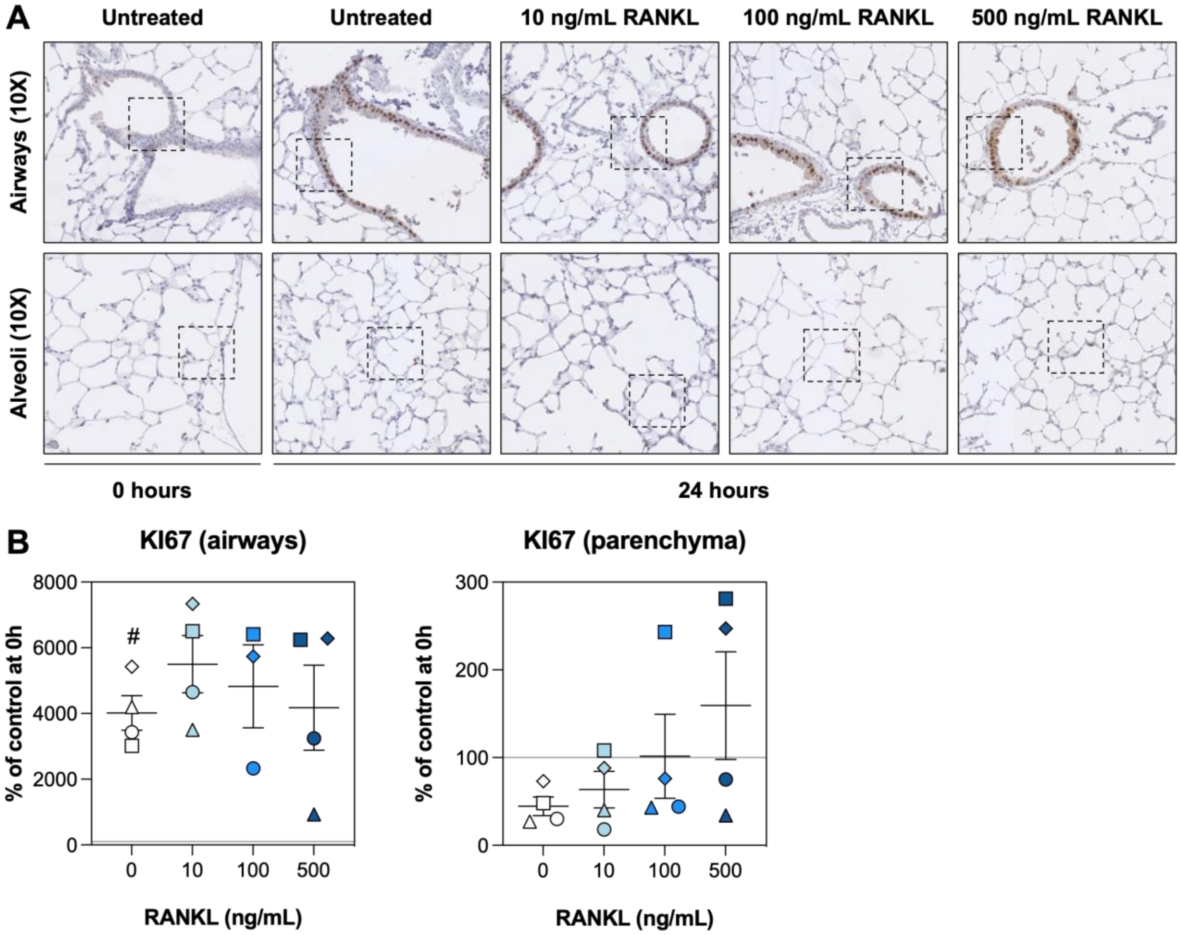

Supplementary figure S2. KI67 stainings. Slices were sampled after slicing (0 h) and after 24 h of incubation without or with 10, 100, or 500 ng/mL RANKL ($n = 4$). KI67 stainings (a) were carried out to visualize proliferating cells (a), and subsequent algorithmic analysis (b) revealed the degree of cell proliferation. (# indicates $p < .05$ between untreated slices at 0 and 24 h)

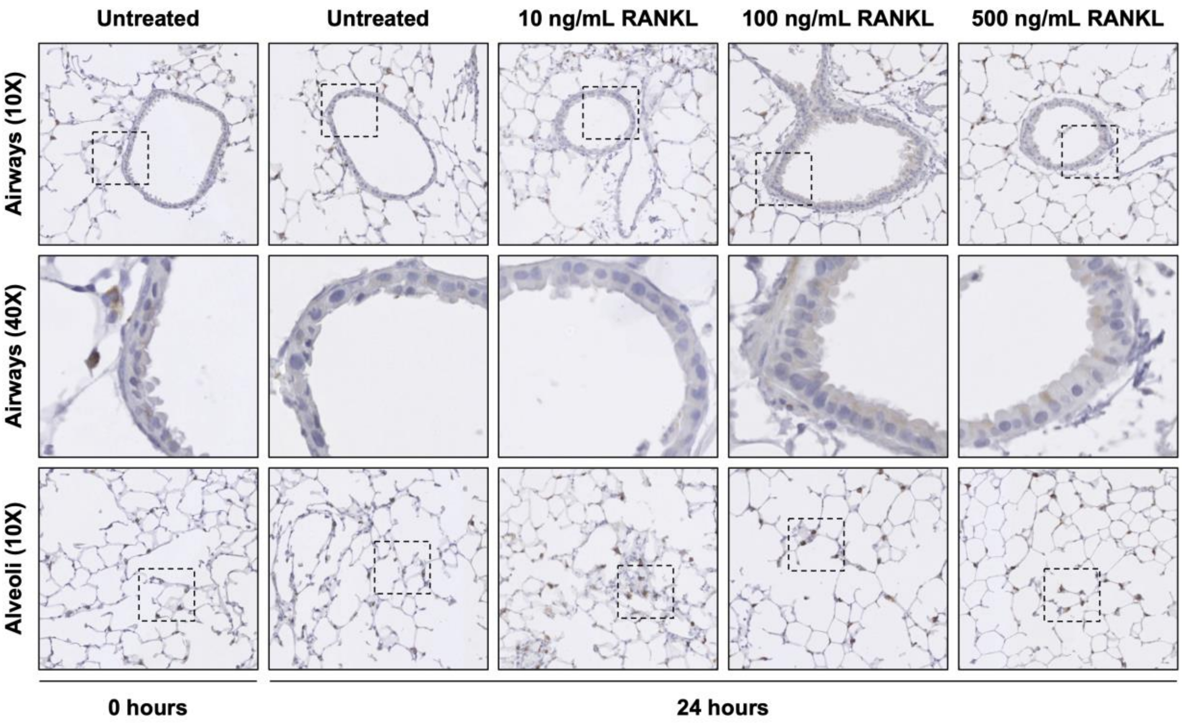

Supplementary figure S3. SFTPC stainings. Slices were sampled after slicing (0 h) and after 24 h of incubation without or with 10, 100, or 500 ng/mL RANKL (n = 4). SFTPC stainings were subsequently conducted to visualize ATII cells.

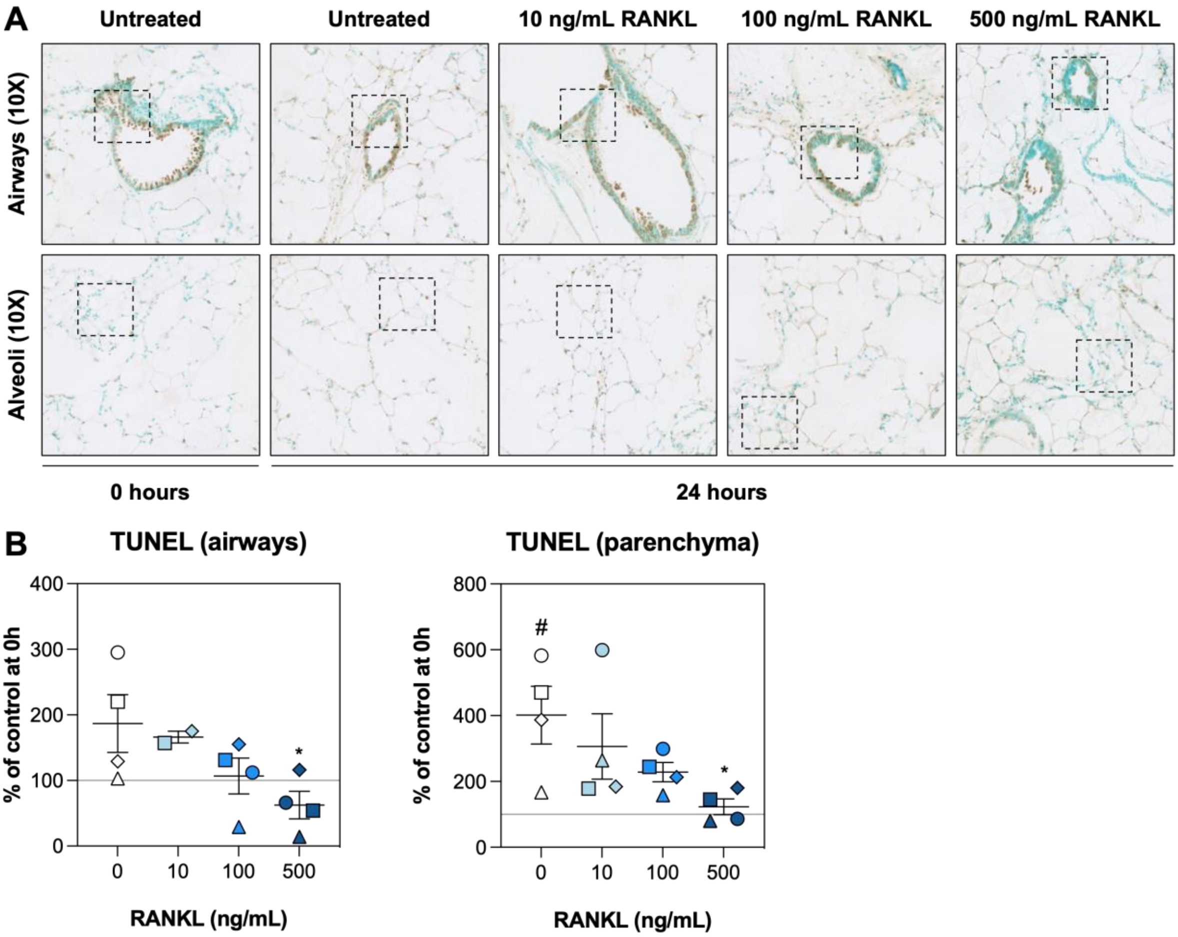

Supplementary figure S4. TUNEL stainings. Slices were sampled after slicing (0 h) and after 24 h of incubation without or with 10, 100, or 500 ng/mL RANKL (*n* = 4). TUNEL stainings (a) were carried out to visualize DNA fragmentation, after which algorithmic analysis (b) revealed the extent of DNA fragmentation. (# indicates $p < .05$ between untreated slices at 0 and 24 h; * indicates $p < .05$ between untreated slices and slices treated with exogenous RANKL at 24 h)
